# Beyond intentions: a critical narrative review of accreditation’s planned and emergent impact on medical schools

**DOI:** 10.1007/s10459-026-10501-7

**Published:** 2026-01-24

**Authors:** Do-Hwan Kim, Roghayeh Gandomkar, Hyo Hyun Yoo, David Rojas

**Affiliations:** 1 Department of Medical Education, College of Medicine, Korea University, 73, Goryeodae-ro, Seongbuk-gu, Seoul, 02841 Republic of Korea; 2 Departments of Medical Education, School of Medicine, Tehran University of Medical Sciences, Tehran, Iran; 3 Health Profession Education Research Center, Tehran University of Medical Sciences, Tehran, Iran; 4 Department of Medical Education, College of Medicine, Jeonbuk National University, Jeonju, Republic of Korea; 5 The Wilson Centre, Temerty Faculty of Medicine, University of Toronto, Toronto, Ontario Canada; 6 Department of Obstetrics and Gynaecology, Temerty Faculty of Medicine, University of Toronto, Toronto, Ontario Canada

**Keywords:** Accreditation, Medical education, Complexity, Quality assurance, Quality improvement

## Abstract

As medical schools rapidly expand worldwide, accreditation has become a key mechanism for ensuring and improving the quality of medical education. However, its effectiveness remains unclear due to conflicting research results and limited empirical evidence. Therefore, the aim of this critical narrative review is to synthesize and analyze the intended (planned) and unintended (emergent) impacts of medical school accreditation. We conducted a critical narrative review by systematically searching five databases: MEDLINE, EMBASE, ERIC, Global Index Medicus, and ProQuest. The literature search was conducted between October and November 2024. Thirty-two empirical studies on the impact of medical school accreditation were included. Following a subjectivist orientation, we combined deductive categorization and inductive thematic analysis for analysis. Three researchers conducted repeated coding and data extraction through a consensus process, identifying both planned and emergent processes and outcomes. The planned processes included governance reform, faculty and student engagement, infrastructure investment, data monitoring and sharing, structured curriculum reform, and the introduction of program evaluation of quality improvement systems. These contributed to improved student outcome, curriculum refinement, and enhanced institutional awareness. However, emergent processes were identified, such as navigating conflicting demands, strategically controlling data disclosure, and adapting to changes in standards. These led to emotional ambivalence among stakeholders, resources intensification, and short-term strategizing that hindered sustainable reform. These results challenge the assumption that accreditation operates linearly, emphasizing that its impact on medical schools is accompanied by both intended and unintended effects. Policymakers and institutions must recognize the dual nature of accreditation, promote sustainable engagement, and proactively address unexpected outcomes to ensure that accreditation aligns well with long-term educational goals.

**Supplementary information:**

The online version contains supplementary material available at https://doi.org/10.1007/s10459-026-10501-7.

## Introduction

The global number of medical schools has surged over the last decade, sparking widespread concern about educational quality. In 2014, the number stood at roughly 2600 (Duvivier et al., 2014), climbing to 3300 by 2020 (Bedoll et al., 2021), and exceeding 4000 by 2024 (WFME, 2024). However, this rapid growth has often involved the creation of new schools lacking robust institutional or financial support, contributing to significant variation in the quality of medical education (Avena et al., 2025; Tsinuel et al., 2016). Coupled with the increasing cross-border mobility of healthcare workers, this variation means that quality issues in one country can have global implications. Within this context, there has been growing consensus on the need for accreditation as a regulatory mechanism to ensure minimum standards in medical education and build cross-national trust in the competence of medical graduates (Shiffer et al., 2019). As of 2025, 202 accrediting organizations were operating across 152 nations, reflecting the global establishment of mechanisms to manage and enhance the quality of medical education (FAIMER, 2025).

In response to growing global concerns about educational quality, accreditation has evolved to fulfill a dual purpose: ensuring minimum standards through quality assurance (QA) and fostering enhancement through quality improvement (QI) (Ahn, 2020; Amaral & Norcini, 2023; Harvey & Williams, 2010). This dual orientation is clearly reflected in the formal definition of accreditation (Taber et al., 2020). Most accreditation systems incorporate common core components to achieve these objectives, such as the clearly defined evaluation standards, predetermined protocols, and regular cycles of review and monitoring (Boulet & van Zanten, 2014; Greenfield & Braithwaite, 2008; Taber et al., 2020). Agencies commonly operationalize QA by withholding accreditation from non-compliant institutions, while advancing QI by disseminating exemplary practices to promote collective advancement.

While accreditation is intended to promote quality and improvement in Undergraduate Medical Education (UME), questions persist about its real-world effectiveness. Empirical studies yield conflicting results regarding its effectiveness. Some research indicates that accreditation has facilitated substantive curricular reforms (Blouin et al., 2018; Kassebaum, Cutler, et al., 1997), yet other accounts often describe these changes as symbolic, transient, and lacking long-term sustainability (Alrebish et al., 2017; Gul et al., 2022; Roy et al., 2020). Likewise, while accreditation has been credited with enhancing faculty engagement in teaching and raising awareness of educational quality (Arja et al., 2024; Knollmann-Ritschel et al., 2017), it has also been criticized for imposing substantial administrative burdens that contribute to faculty burnout and decreased educational engagement (Caverzagie et al., 2017; Maccarrick, 2009; Yengo-Kahn et al., 2017).

Beyond the inconsistency in existing findings, a limited empirical foundation further impedes our ability to determine whether accreditation achieves its intended outcomes. A scoping review highlighted that, although research on UME accreditation has expanded since the 1990s, the evidence base remains disproportionately small relative to the global investment in accreditation systems (Tackett et al., 2019). Only about 10% of the studies included were empirical, and most were conducted in high-income countries. Moreover, literature frequently assumes the inherent value and effectiveness of accreditation, with few studies critically examining its limitations or potential drawbacks. These gaps underscore the necessity of examining how accreditation functions across diverse settings. Its effects are not universally consistent, and perspectives from low- and middle-income countries are essential for a more holistic understanding (Alismail et al., 2024; Vento, 2025).

To critically evaluate the impact of accreditation, it is not enough to address the empirical limitations of prior research; we must also interrogate the underlying assumptions that shape how accreditation is conceptualized and studied (Rashid, 2023). One such assumption is the conventional framing of accreditation as a predictable, linear process. This perspective, however, fails to recognize that a medical school is a Complex Adaptive System (CAS) (Frye & Hemmer, 2012; Rojas et al., 2018). As a CAS, it comprises numerous interconnected agents, such as faculty, students, and administrators, whose non-linear interactions create feedback loops, leading to often unpredictable and emergent behaviors (Ogden et al., 2023). Accreditation, in this context, acts not as a simple directive but as a significant intervention or pressure applied to this complex system.

Consequently, viewing accreditation through a complexity lens reveals the inadequacy of a linear approach to evaluation focused solely on intended goals. The very nature of a CAS dictates that any intervention will inevitably produce both planned consequences and unintended, emergent ones (Braithwaite et al., 2018). Building on this recognition, an evaluation framework was proposed to capture this dynamic by incorporating both planned and emergent dimensions, highlighting that in complex programs, it is essential to examine not only intended processes and outcomes, but also those that emerge unexpectedly (Haji et al., 2013). Within this framework, *planned processes* and *outcomes* represent the intentional, organized activities and their anticipated results, premised on a linear logic designed to meet accreditation standards. Conversely, *emergent processes* and *outcomes* encompass the context-specific, adaptive practices and unintended local and system-level patterns that organically arise as institutions navigate accreditation demands amid the complex nature of institutional dynamics. This conceptual distinction enables evaluators to move beyond the initial planned theory and engage with an emergent theory that seeks to answer, “Why is it happening?”. Although some studies have noted unintended effects of accreditation (Blouin et al., 2018; Girotto et al., 2025), these observations are often treated as peripheral observations rather than central subjects to analysis. Such a tendency reflects the enduring influence of a linear framework, one that assumes adherence to standards and procedures will predictably and reliably lead to QA and QI.

However, when viewed through the lens of complexity theory, educational systems are not static or predictable, and the effects of accreditation cannot be reduced to a simple input–output relationship (Jorm & Roberts, 2018). It becomes essential to acknowledge that accreditation, even when well-intended, may produce unintended consequences in practice: some of which may be positive, others negative, and many of which may deviate significantly from the original goals envisioned by accreditation agencies (Cartmill et al., 2024). Given this context, this critical narrative review seeks to address the following question: What are the intended (planned) and unintended (emergent) impacts of accreditation in medical schools, considering both processes and outcomes?

## Methods

This study is a critical narrative review based on literature collected through a systematic search strategy. Unlike systematic reviews, which are rooted in an objectivist orientation, this approach was selected for its suitability in synthesizing heterogeneous evidence to identify emergent patterns inherent in complex systems (Varpio et al., 2024). This review seeks a deeper and more nuanced understanding by examining the impact of accreditation on medical schools from multiple perspectives (Sukhera, 2022). Specifically, ‘multiple perspectives’ refers to our analysis of the viewpoints of stakeholders reported in the literature, the analytical perspectives adopted by the original authors, and our own interpretations as researchers with experience in accreditation. This multi-layered approach allowed us to critically engage with the evidence and its underlying assumptions.

To achieve this, a narrative review with a subjectivist orientation was conducted, wherein the researcher does not strive to eliminate subjective perceptions of the phenomenon but rather actively engages with their own experiences, beliefs, and perspectives as analytical tools for interpreting the literature (Kahlke et al., 2023a). We operationalized this by leveraging our team’s positionality as an interpretive lens. For example, we questioned idealized reports of ‘structured curriculum reform,’ using our insider knowledge of diverse institutional contexts to identify adaptive behaviors like ‘strategic short-termism.’ This approach is central to the critical narrative review, which specifically aims to critically examine taken-for-granted assumptions about the phenomenon, thereby offering a new lens through which it can be understood (Kahlke et al., 2023b).

### Search strategy

The literature search was conducted between October and November 2024, across Ovid MEDLINE, EMBASE, ERIC (Educational Resources Information Centre), Global Index Medicus, and ProQuest Dissertations & Theses. A comprehensive search strategy was developed in collaboration with a professional librarian to ensure thorough retrieval of relevant studies. The full, detailed search strategies for all five databases, including all search terms and Boolean operators, are provided in Appendix 1. The librarian collaborated with the research team, reviewing pre-identified relevant studies to establish appropriate search terms and database selection. A preliminary search was first conducted, allowing for refinement of search terms, which were then applied in the final search. No restrictions were placed on publication year.

### Study selection

The study selection process was conducted in three stages: the initial screening of titles and abstracts (Stage 1), the second-level screening for eligibility (Stage 2), and the full-text review (Stage 3). The specific inclusion and exclusion criteria applied throughout this process are detailed in Table 1.

**Table 1:** Inclusion and exclusion criteria

Category	Inclusion criteria	Exclusion criteria
Language	Articles published in English.	Articles published in non-English languages
Disciplinary Focus	Studies focused on medical school accreditation within the context of medical education	Studies focused on biomedical science topics (e.g., gene expression regulation) or non-medical school accreditation (e.g., hospitals, laboratories)
Educational Level	Studies addressing undergraduate medical education (UME)	Studies addressing stages of education beyond undergraduate medical education (e.g., GME, CME).
Professional Domain	Studies related to medical health professions education.	Studies related to non-medical health professions education (e.g., dentistry, nursing).
Study Type	Peer-reviewed original studies that presented empirical data (quantitative or qualitative)	Studies that did not present original empirical data (e.g., reviews, editorials)

In Stage 1, 10,523 unique articles were screened by a single author (DHK). The goal was twofold: to efficiently exclude studies that were clearly irrelevant using strict, pre-defined exclusion criteria, while adopting an intentionally inclusive approach to minimize the risk of prematurely excluding relevant studies. Applying these criteria, which encompassed language, disciplinary focus, educational level, and professional domain, resulted in the exclusion of non-English studies, primarily due to limitations in the research team’s collective language proficiency and the finite resources available for translation. To ensure the reliability of this extensive initial screening, several support mechanisms were implemented. Prior to the screening, the full research team collaboratively developed and finalized the explicit exclusion criteria through multiple rounds of discussion to ensure a shared understanding. The research team held regular meetings where the primary screener reported on progress, and the team reviewed batches of excluded studies to confirm the correct application of the criteria and to ensure transparency and consensus throughout the process.

In Stage 2, articles that passed the initial screening underwent a second level of review by three authors (DR, RG, and DHK). Each author independently assessed the titles and abstracts to determine their eligibility for full-text review. After making individual judgments, the three authors then convened to discuss their judgments and resolve any discrepancies, reaching a consensus on the final list of articles to be reviewed in full.

In Stage 3, the articles deemed potentially eligible underwent a full-text review to confirm they met all criteria, specifically targeting peer-reviewed original studies with empirical data (either quantitative or qualitative) on the impact of accreditation on medical schools, including their members such as students and faculty. Of the studies selected for full-text review, 20% were read in full by three authors (DR, RG, and DHK) to ensure consistency in applying the inclusion and exclusion criteria and to reach agreement on data extraction categories. The remaining studies were then divided among the three authors for review. During the article retrieval process, 2 articles were excluded as their full texts were unavailable. The remaining 39 articles were reviewed in full, leading to further exclusion of 6 articles: 2 were duplicates and 4 did not present original empirical data. This resulted in the final 32 articles included in this review.

### Data extraction and synthesis of results

The three authors independently carried out data extraction using a shared set of criteria and categories that had been refined through iterative discussions and consensus-building. The first author compiled the extracted data and reviewed it to identify any ambiguous content or elements warranting further discussion. These cases were addressed through routine meetings in which the team worked collectively to resolve discrepancies. Extracted items included the publication details (author, year), contextual factors (country/region, income setting), methodological characteristics (study design, primary data source), the primary findings, the accreditation components associated with those findings, and their preliminary classification into four analytic categories: Process vs. Outcome and Planned vs. Emergent.

To explain the impact of accreditation on medical schools, both inductive and deductive analytical strategies were employed. For the deductive phase, definitions of the four categories: Planned Process, Planned Outcome, Emergent Process, and Emergent Outcome, were developed using Haji et al.’s evaluation model as a foundational framework and incorporating insights from literature on medical education as a complex system. Based on these definitions, the researchers categorized the key findings of each study, meeting regularly to discuss items that were difficult to classify or subject to disagreement. The inductive analysis aimed to identify recurring themes and patterns within each category using thematic analysis (Kiger & Varpio, 2020). While general accreditation criteria informed the thematic coding, the analysis of emergent components intentionally adopted a broad perspective that encompassed a wide range of individual and organizational behaviors. During the analysis, we accounted for the potential influence of study design and context. To operationalize this, our data extraction form included fields designed to relate each finding’s classification (Planned Process, Planned Outcome, Emergent Process, or Emergent Outcome) to its underlying study design (e.g., qualitative, quantitative) and context (e.g., income-setting). To enhance the trustworthiness of the analysis, we adopted an iterative approach that combined independent coding with successive rounds of team-based interpretive discussion. This process allowed us to challenge initial impressions, reconcile divergent interpretations, and ensure that thematic categories remained grounded in the data.

### Reflexivity

We adopted a subjectivist orientation, using our collective accreditation experience as an analytic lens. Our team comprises four medical education researchers who all share a deep ‘insider’ perspective, having experienced accreditation as both auditees (e.g., site visit preparation, institutional self-study, program evaluation) and assessors (e.g., standards committees, site visits, national accreditation committee). This shared duality positioned us to analyze the literature through a lens of inherent tension: understanding the ‘planned’ intentions of standards (from our assessor roles) while recognizing the complex, contextual, and ‘emergent’ realities of their implementation (from our auditee roles).

Our positionality is intentionally diverse. DHK brings insights from three private medical schools, illustrating organizational culture, leadership, curricular history, hospital-university dynamics, institutional scale, and available resources mediate institutional responses to identical standards. HHY offers a 10-year perspective from a standards committee and concurrent self-study leadership in a national medical school, foregrounding trust among standards, survey practice, and institutional candor as a precondition for improvement. RG provides a crucial non-Western viewpoint from an LMIC (Iran), grounding the analysis in resource-limited contexts by sensitizing the team to feasibility, equity, and policy constraints. DR shares this dual practitioner role through his leadership in program evaluation for accreditation in Canada, while also bringing a formal methodological expertise as a scientist whose research focuses on complexity theory and unintended consequences. Our analytical process involved iterative reflexive dialogue, where our collective practitioner experiences of ‘strategic adaptations’ were conceptualized through the theoretical language of ‘emergence’. This synthesis of shared, multi-context, dual-role practitioner knowledge with systems-level methodological expertise is how we operationalized our subjectivist stance, enabling a critical analysis of the gap between accreditation’s linear intentions and its complex, lived realities.

### Ethical approval

As this study involved a review of existing published literature, ethical approval was not required.

## Results

**Fig. 1:**
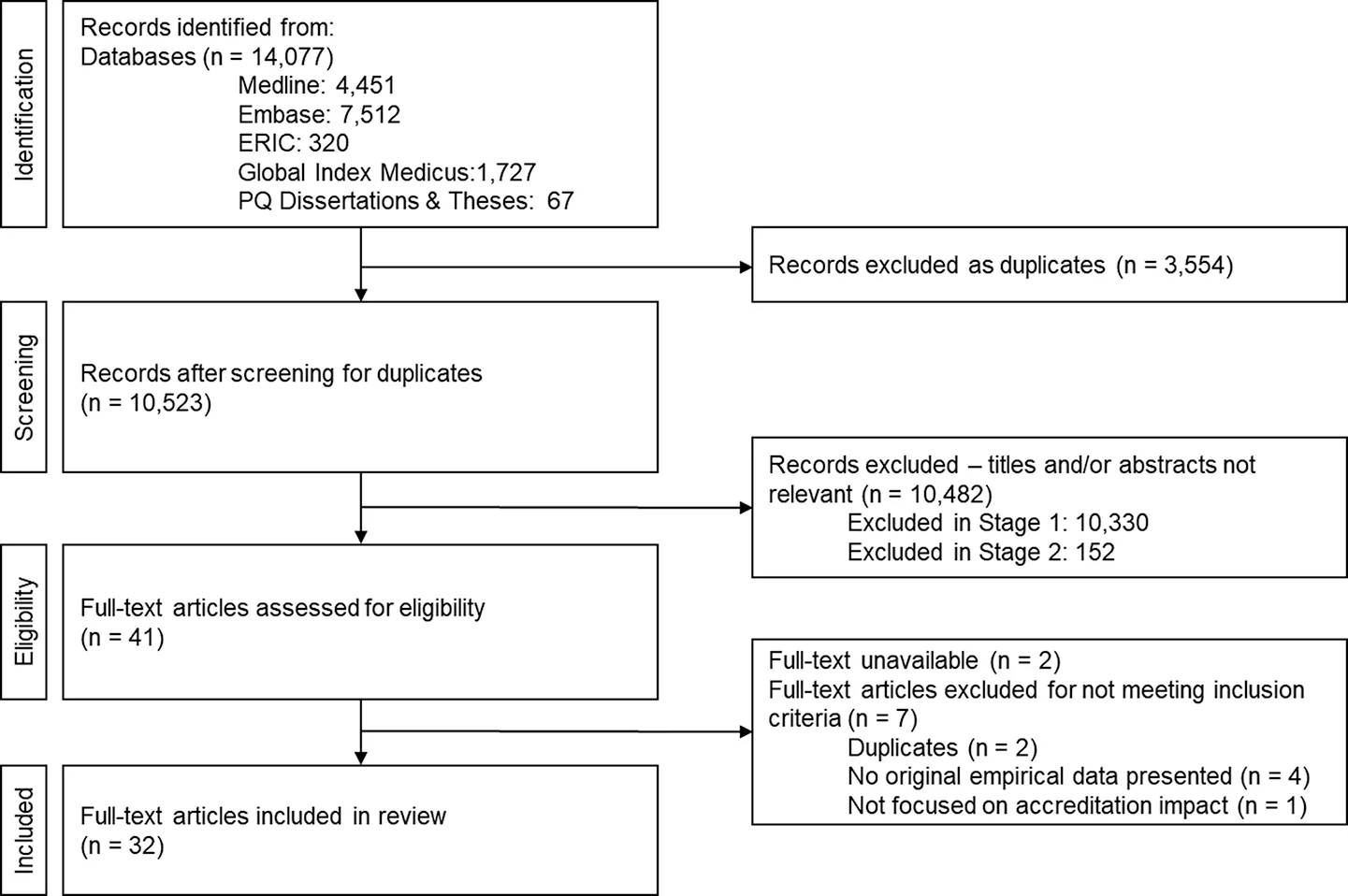
Study selection process

The initial database search yielded a total of 14,077 articles. After removing duplicates, 10,523 unique articles remained. Following the first screening phase, 193 articles were retained. In the second stage, these 193 articles were reviewed in greater detail, resulting in a final selection of 41 articles deemed eligible for full-text review. Of these, 32 articles were included in the final review (Fig. 1).

### Study characteristics

The characteristics of the 32 included studies are summarized in Appendix 2. The articles were published between 1997 and 2024, with a notable increase in publications in the last decade (*n* = 22, 69% published since 2015). The studies originated from a diverse range of economic contexts: 18 (56%) were from High-Income countries (predominantly the United States and Canada), 5 (16%) from Upper-Middle-Income, 3 (9%) from Lower-Middle-Income, and 6 (19%) from varied settings. By country or scope, the United States was most common (*n* = 11, 34%), followed by multi-country (*n* = 4, 13%), Canada (*n* = 4, 13%), China (*n* = 3, 9%), and global scope (*n* = 3, 9%). Regarding study design, 11 were quantitative (34%), 10 qualitative (31%), 6 mixed-methods (19%), and 5 descriptive case studies (16%).

### Influence of context and study design on findings

The context and design of the included studies appeared to shape the focus of their findings. A distinct contextual pattern was observed across study settings. Studies with a ‘Global’ scope (*n* = 3) exclusively utilized large-scale databases to assess planned outcomes, such as the performance of international medical graduates on standardized examinations (e.g., Tackett et al., 2021; van Zanten et al., 2022). In contrast, studies from High-Income Countries (*n* = 18), particularly the United States and Canada, were the primary source for identifying emergent impacts, often describing institutional adaptations to accreditation standards (e.g., Blouin et al., 2018; Schnabl, 2012). Studies from Lower and Upper-Middle-Income Countries (*n* = 8) tended to focus on planned impacts, including evaluations of accreditation agency activities (e.g., Altıntaş et al., 2024) or the impact of standards implementation (e.g., Villamor & Reyes, 2016).

Study design was also a determinant of the findings. Quantitative studies (*n* = 11), which typically analyzed databases or surveys, concentrated on measuring predefined Planned Outcomes (8 of 11 studies), such as the relationship between accreditation status and student examination scores (e.g., Roy et al., 2020; van Zanten, 2015). Conversely, qualitative studies (*n* = 10), using methods like interviews and focus groups, were the main source for inductively derived findings. These studies frequently captured Emergent Processes and Outcomes (identified in 8 of 10 studies), offering insights into the challenges and tensions perceived by stakeholders (e.g., Khan et al., 2020; Onyura et al., 2024).

Following the full-text review, we identified six planned processes and three planned outcomes associated with accreditation. Additionally, three emergent processes and three emergent outcomes were identified as unplanned but consequential effects. An overview of these components is provided in Table 2, and each is further elaborated in the subsequent sections.

**Table 2:** Planned and emergent processes and outcomes of accreditation in undergraduate medical education

	Planned	Emergent
Process	PP1. Change in Governance and Organizational Structure PP2. Faculty and Student Engagement with the institution PP3. Investment in Infrastructure and Resources PP4. Data Collection, Monitoring, and Sharing PP5. Structured Curriculum Reform PP6. Program Evaluation and Continuous Quality Improvement	EP1. Navigating Conflicting Accreditation Demands EP2. Strategic Control of Data Disclosure EP3. Adapting to Evolving Accreditation Standards
Outcome	PO1. Enhanced Student Outcomes PO2. Refined Curriculum PO3. Increased Institutional Awareness	EO1. Emotional Ambivalence Toward Accreditation EO2. Resource Intensification and Opportunity Costs EO3. Strategic Short-Termism Under Pressure

### Planned processes (PP)

We defined the *planned process* as the intentional and organized activities undertaken by a program in pursuit of accreditation success. Within this domain, we identified six thematic categories that reflect the various ways in which accreditation shapes formalized planning and execution within institutions.

#### PP1. Change in governance and organizational structure

A consistent finding across several studies was the shift toward more formalized governance and structural reorganization in response to accreditation demands. Institutions frequently established new entities such as curriculum and assessment committees, as well as QA and QI units (Arja et al., 2024; Blouin et al., 2018). In addition to creating new structures, institutions also formalized internal processes by clearly delineating roles and responsibilities within various committees (Altıntaş et al., 2024), and implementing new or revised institutional policies (Arja et al., 2021).

#### PP2. Faculty and student engagement with the institution

Accreditation was often cited as a mechanism for fostering greater institutional engagement among faculty and students. Faculty members were increasingly involved in institutional decision-making and contributed actively to reform initiatives (Arja et al., 2021, 2024; Blouin et al., 2018). In some cases, institutions appointed deans or associate deans specifically for curriculum development and evaluation, underscoring the importance of academic leadership in sustaining faculty participation (Arja et al., 2024). At the same time, student engagement also intensified. Many schools expanded student representation within governance bodies (Altıntaş et al., 2024), and strengthened student support services, including the introduction of formal counseling programs (Altıntaş et al., 2024; Arja et al., 2024).

#### PP3. Investment in infrastructure and resources

Accreditation preparation often prompted significant institutional investments in infrastructure and resources. These included enhancements to library collections, adoption of digital software systems, and upgrades to clinical, laboratory, and physical facilities (Altıntaş et al., 2024; Hedrick et al., 2019; Villamor & Reyes, 2016). Many institutions also established dedicated staff positions to manage accreditation processes (Blouin et al., 2018; Chandran et al., 2013). These efforts were often supported by external funding opportunities, such as foundation grants, mobilized in response to accreditation demands (Kassebaum, Cutler, et al., 1997).

#### PP4. Data collection, monitoring, and sharing

Accreditation processes motivated medical schools to develop or enhance data infrastructures for collection, analysis, and reporting. Institutions gathered a broad array of data, including curricular content, student and faculty feedback, program outcomes, and alumni perceptions (Arja et al., 2024; Blouin et al., 2018). To manage these activities, many schools formed dedicated committees to oversee monitoring and documentation efforts (Arja et al., 2021). Furthermore, they utilized digital tools such as dashboards, learning management systems (LMSs), and institutional websites to promote data transparency and facilitate information sharing (Arja et al., 2024; Blouin et al., 2018; Chandran et al., 2013; Hedrick et al., 2019).

#### PP5. Structured curriculum reform

Accreditation processes frequently served as a catalyst for curriculum reform, prompting changes that were less the result of internal initiative and more aligned with strategic responses to the standards (Geffen et al., 2014; White et al., 2013). To meet specific accreditation requirements, such as centralized curriculum oversight, schools restructured key governance bodies, including the appointment of deans as chairs of curriculum committees (Chandran et al., 2013) and established ad hoc working groups jointly led by faculty and students (Scott et al., 2020). These reforms were further supported through collaboration between curriculum evaluation units and committees, leading to systematic and ongoing course review processes aimed at driving curricular reform (Ward et al., 2018).

#### PP6. Program evaluation and continuous quality improvement (CQI)

A common organizational response to accreditation was the development or formalization of CQI processes. Many schools established dedicated CQI committees (Arja et al., 2021) and appointed staff specifically for quality functions (Blouin et al., 2018). Some also implemented structured models such as the Plan-Do-Study-Act (PDSA) cycle to guide improvement efforts (Hedrick et al., 2019; Ward et al., 2018). CQI activities were further supported through the integration of software platforms and their incorporation into routine academic operations (Hedrick et al., 2019). Additionally, schools engaged in external benchmarking, drawing insights from peer institutions and published literature to refine their quality strategies and adopt best practices (Arja et al., 2024).

### Planned outcomes (PO)

We defined *planned outcomes* as the specific and tangible results that arise from the deliberate execution of planned processes. These outcomes are typically aligned with institutional goals and accreditation criteria, serving as concrete evidence of compliance and progress. Our analysis identified three themes within this category.

#### PO1. Enhanced student outcomes

Accreditation has been associated with improvements in student outcomes across both national and international contexts, particularly at the reaction and learning levels of educational evaluation. At the reaction level, students reported more positive perceptions of their learning environments following accreditation processes (Arja et al., 2024). With regard to learning, multiple studies, particularly those examining international medical graduates (IMGs), indicate that graduates from accredited institutions achieve higher first-attempt pass rates on the USMLE (van Zanten, 2015; van Zanten et al., 2022, 2012) and are more likely to obtain ECFMG certification (Tackett et al., 2021). Similar trends have been documented in countries such as Mexico (Gaxiola-García et al., 2020) and China (You et al., 2024), where accreditation has been linked to improved performance on national licensing exams.

#### PO2. Refined curriculum

Accreditation has also led to tangible improvements in curriculum structure and delivery. Changes included the integration of basic and clinical sciences and increased exposure to ambulatory and primary care (Kassebaum, Cutler, et al., 1997), revision of their institutional learning objectives (Arja et al., 2024) and adoption of student-centered instructional methods such as small-group activities (Altıntaş et al., 2024). In some institutions, these reforms extended further, with innovative approaches like discovery learning implemented throughout the preclinical curriculum and sustained over time (White et al., 2013).

#### PO3. Increased institutional awareness

Accreditation processes encouraged institutions to develop a deeper awareness of their organizational realities. The self-study phase, particularly in preparation for site visits, served as a meaningful opportunity to revisit foundational educational philosophies (Geffen et al., 2014), and to acknowledge the importance of both interdepartmental and intradepartmental collaboration (Muhtadi, 2013). This process also helped overcome internal resistance to change (Muhtadi, 2013) and reflection on issues such as diversity (Salcido, 2013) and social accountability (Koepke et al., 2020).

### Emergent processes (EP)

We defined *emergent processes* as context-specific, adaptive, and responsive institutional practices that arise in response to unanticipated challenges or opportunities encountered during the accreditation process. Unlike planned processes, these are not predetermined but arise organically as institutions navigate the accreditation landscape. Within this category, we identified three themes.

#### EP1. Navigating conflicting accreditation demands

Accreditation processes often revealed underlying tensions between accreditation standards and institutional or regulatory constraints. At the institutional level, conflicts frequently arose from misalignment between accreditation requirements and pre-existing regulations, organizational priorities, or resource realities (Gul et al., 2022). In some cases, schools found it difficult to reconcile the competing priorities of affiliated hospitals and medical education programs. Accreditation also highlighted tensions between short-term compliance efforts and broader, long-term goals such as educational quality enhancement (Alrebish et al., 2017). At the regulatory level, tensions arose when requirements for faculty diversity, such as those from the LCME, encountered legal prohibitions on race- or gender-based hiring considerations (Schnabl, 2012).

#### EP2. Strategic control of data disclosure

While accreditation promotes transparency and accountability, several studies identified persistent constraints in actual data sharing. Within institutions, faculty and stakeholders often lacked access to important metrics such as USMLE scores and residency match rates (Arja et al., 2024). In some instances, medical schools limited the dissemination of certain evaluation results, either withholding them from student leaders or opting not to submit internal data to accreditation agencies (Onyura et al., 2024). At the broader level, research on Chinese medical schools highlighted considerable variability in the extent of public data disclosure, with transparency differing by information type and accreditation status (Li et al., 2022).

#### EP3. Adapting to evolving accreditation standards

Accreditation often demanded ongoing adaptation as standards evolved in definition, focus, and implementation. This theme was most apparent in systems with established accreditation practices, such as the LCME in the U.S. For instance, one study documented how definitional ambiguities and varying emphases among surveyors influenced institutional responses and perceptions of noncompliance (Kassebaum, Eaglen, et al., 1997). Another study demonstrated that changes to LCME criteria altered the focus and frequency of severe accreditation decisions (Hunt et al., 2012). As standards became less prescriptive, institutions were required to interpret compliance expectations themselves, prompting broader organizational alignment efforts (Knollmann-Ritschel et al., 2017).

### Emergent outcomes (EO)

We defined *emergent outcomes* as results that arise unintentionally from emergent processes; such outcomes are developments that were not explicitly anticipated during the planning phase but become apparent as the accreditation practice unfolds. Our analysis identified three themes within this category.

#### EO1. Emotional ambivalence toward accreditation

Accreditation generated a complex mix of emotional responses among institutional stakeholders. On one hand, it functioned as a motivating force that generated momentum for long-overdue reforms (Arja et al., 2021). In this sense, accreditation served as a catalyst, accelerating reforms that may have otherwise been delayed or deprioritized (White et al., 2013). At the same time, many also described the process as ‘painful’. Faculty members often reported feelings of fatigue, diminished morale, and in some cases, burnout related specifically to accreditation (Blouin et al., 2018; Muhtadi, 2013; White et al., 2013).

#### EO2. Resource intensification and opportunity costs

Accreditation also gave rise to substantial, and often unanticipated, resource burdens. In some regions, the cost of site visits alone ranged from $100,000 to $150,000 (Arja et al., 2021), with additional resources required for preparation, infrastructure improvement, and faculty/staff time (Muhtadi, 2013). Over successive cycles, institutions faced rising accreditation costs along with opportunity costs, as resources were increasingly diverted from innovative or long-term initiatives to address immediate compliance demands (Blouin et al., 2018).

#### EO3. Strategic short-termism under pressure

Accreditation’s influence also led some institutions to adopt short-term compliance strategies. For instance, some schools adjusted their program evaluations to highlight positive indicators, placing greater emphasis on summative outcomes while neglecting formative or process-oriented measures (Onyura et al., 2024). In other cases, accreditation was treated as a periodic obligation rather than an ongoing commitment, with institutions reverting to less effective practices after accreditation was achieved (Khan et al., 2020). Performance trends in student data also revealed cyclical patterns, with outcomes peaking around site visits and declining in between (Roy et al., 2020).

## Discussion

Adopting a complexity systems perspective, this review analyzed how accreditation shapes institutional behavior in medical education through both planned and emergent processes and outcomes. By structuring its analysis across these dual axes, the study moved beyond normative goals like QA and QI to reveal how accreditation functions in practice, often in ways that are not explicitly anticipated. While planned components generally contributed to improvements in educational quality, emergent components exposed the trade-offs institutions make to satisfy accreditation demands, sometimes producing unintended consequences at odds with accreditation’s intended aims. A pattern was observed where this focus was shaped by study design and context: quantitative studies, particularly those with a ‘Global’ scope using large databases, focused almost exclusively on planned outcomes (e.g., exam scores). In contrast, qualitative studies, often situated in High-Income Countries, were essential for identifying complex emergent processes and unintended outcomes. These findings point to a need for theoretical expansion: planned theory explains design intentions (“Why will it work?”), but emergent theory attends to lived realities (“Why is it happening?”) (Haji et al., 2013). The following discussion develops these two theoretical perspectives and examines the assumptions that underpin each.

### Planned theory and emergent theory of accreditation

#### Planned theory of accreditation

The planned theory of accreditation can be summarized as follows: the introduction of an accreditation system will (1) present standards that articulate the characteristics of a high-quality medical school, (2) encourage institutions to identify opportunities for improvement and take concrete steps to realize them, and (3) ultimately lead to tangible advancements in curriculum and educational outcomes. This theory is grounded in a series of linear assumptions that connect step (1) through (3).

Based on the findings of this study, the planned theory of accreditation appears to hold to a certain extent. For instance, the six planned processes identified in this review align closely with the key domains emphasized in the standards of various accreditation bodies (Benbassat et al., 2022; Hays, 2014; Kassebaum et al., 1998; Sharifi et al., 2021; Tackett et al., 2016). This alignment suggests a functional link between steps (1) and (2) in the planned theory. Although the degree of prescriptiveness may vary across accrediting agencies (Lee et al., 2024; Tempski et al., 2024), the availability of published standards signals the priority areas of accreditation, and this study provides evidence that medical schools respond by taking concrete actions in those areas.

Moreover, by illustrating the connection between steps (2) and (3), the relationships observed between specific planned processes and outcomes reinforce the plausibility of the planned theory. For example, increased faculty and student engagement is likely to contribute to improved student outcomes (Blouin, 2020; Burk-Rafel et al., 2020; Steinert et al., 2024); institutional investment in program evaluation and continuous quality improvement may facilitate curriculum reform (Bendermacher et al., 2020; Stalmeijer et al., 2023); and systematic collection and analysis of curriculum-related data can enhance institutional understanding of their educational programs (Cook et al., 2023; Lomis et al., 2021). These findings suggest that, when implemented purposefully, planned processes can yield tangible, intended impacts.

#### Emergent theory of accreditation

**Fig. 2:**
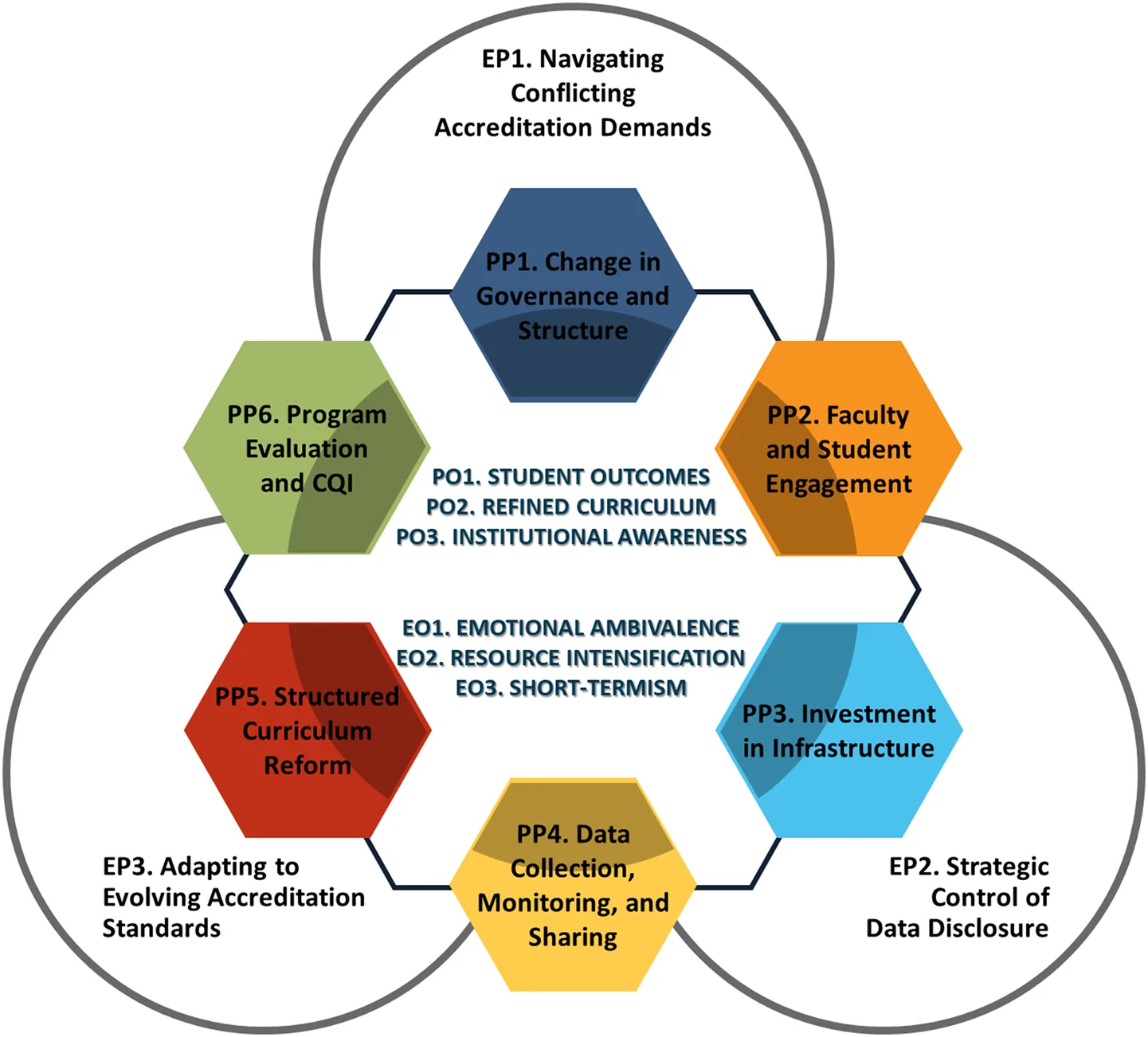
Interplay of planned and emergent components in accreditation impact. This figure presents six planned processes (PP; hexagons) that serve as the principal means by which accreditation influences medical schools. These planned processes are positioned alongside three emergent processes (EP; outer circles), indicating their potential to interact and shape one another. Collectively, these elements contribute to the generation of three planned outcomes (PO) and three emergent outcomes (EO), illustrated at the center of the diagram. Abbreviations: PP = planned Process; PO = planned Outcome; EP = emergent Process; EO = emergent outcome

The planned theory outlined earlier demonstrates accreditation’s intended mechanism by illustrating the processes designed to produce specific outcomes under ideal conditions. Although clearly defining one-to-one relationships between processes and outcomes is challenging, the theory assumes that accreditation achieves its intended goals when multiple processes operate cohesively and centripetally. Conversely, the emergent theory illustrates how this alignment can become destabilized. This theory views the medical school as a CAS, where agents (faculty, students) constantly adapt to external pressures. Therefore, emergent processes are the system’s ‘self-organizing’ and adaptive responses, often operating in the background of planned processes and exerting centrifugal forces that pull institutions away from coherence. This creates an inherent tension between planned intentions and emergent realities. Emergent outcomes, as the unexpected system-level patterns arising from this interaction, generate feedback loops that may introduce vulnerabilities into the achievement of planned outcomes (Fig. 2). As a result, these emergent components constrain the effectiveness of the planned theory, rendering it only partially functional or suboptimal under complex real-world conditions, while also highlighting that success hinges on the system’s adaptive capacity to manage these complex dynamics.

Particularly, the three emergent processes interacted with and weakened the conditions essential for the planned processes to function effectively. For example, efforts to restructure governance and organizational regulations within medical schools (PP1) often encountered friction from broader systemic factors (EP1). Medical schools inherently operate within the dynamics of hospital and higher education frameworks (meso level) and larger national policy structures (macro level) (Jorm & Roberts, 2018; Taber et al., 2010). Yet, accreditation demands were not always compatible with these contextual realities (Tavares et al., 2020). This exemplifies a typical adaptive behavior of a complex system responding to uncertainty and ambiguity; actors navigate the tension between conflicting internal and external demands rather than simply complying with new standards (Khan et al., 2018).

Another example involves the use of institutional data to promote evidence-informed improvements (PP4). While data collection and analysis were intended to support meaningful reform, in practice, data sharing was often strategically limited by medical schools (EP2). This can be understood as an act of self-organization within a low-trust, high-risk environment. To protect themselves from the perceived risks of identification, acknowledgment, and dissemination of areas for improvement, actors locally control information flow, exercising selective control over what is shared with diverse stakeholders (Bland et al., 2000; Talaat et al., 2023). This local adaptation, however, leads to the unintended consequence of impeding the full potential of evidence-informed improvement initiatives (Damodaran et al., 2017; Valentine et al., 2021).

A further example concerns program evaluation and continuous quality improvement (PP6), which were frequently complicated by variability in accreditation standards (EP3). Whereas the planned theory presupposes fixed or stable standards, in practice, these standards undergo temporal changes (diachronic variability) (Hays, 2014; Yoo et al., 2020) and are subject to varying interpretations among evaluators (synchronic variability) (Dos Santos et al., 2017; Due et al., 2019). This turns the standards into a ‘moving target,’ meaning the institution operates not in a predictable, stable environment, but in a dynamic state where continuous adaptation to uncertainty is the norm (Mennin, 2010).

Emergent outcomes arise as unintended consequences at the intersection of planned and emergent processes. Unlike planned outcomes, which are typically framed as desirable achievements, emergent outcomes are characterized by their ambivalence, offering both positive and negative implications for sustainability and long-term impact.

One emergent outcome observed was the rise of emotional strain among faculty and staff actively involved in accreditation (EO1). This dynamic exemplifies a powerful negative reinforcing feedback loop. While increased participation is generally perceived as beneficial, heightened engagement may also bring unintended emotional repercussions, including fatigue, frustration, or burnout. If not balanced with appropriate attention, these negative effects may risk overwhelming the positive contributions of participation. This burnout, in turn, degrades the quality of engagement and fosters cynicism, weakening the momentum for reform in subsequent cycles (Seo et al., 2022).

Another emergent outcome involves the cumulative nature of institutional investments made in response to accreditation pressures (EO2). Given the inherent stakes involved in accreditation, certain expenditures are inevitable; however, without a strategic approach that considers opportunity costs and systemic limitations, institutions may struggle to maintain the improvements achieved during accreditation cycles (Barber et al., 2020; Blouin, 2020).

A further emergent outcome reflects the risk of accreditation-driven reforms becoming overly focused on demonstrable, short-term results (EO3). This exemplifies path dependence, as external pressures often lead to strategic behaviors favoring a local optimum (immediate survival) over a global optimum (long-term innovation). Once entrenched in organizational culture and systems, these strategies lock the system into a cycle of superficial or transient changes, making future continuous quality improvement face greater resistance and cost (Gordon & Cleland, 2021; Swails et al., 2023).

#### Underlying assumptions of accreditation

The emergent theory provides an interpretive lens for understanding a critical question: *Why are planned outcomes not consistently achieved, even when planned processes are implemented?* This question invites a closer examination of the underlying assumptions that connect the elements of the planned theory. Planned theory conceptualizes accreditation as a linear progression from standard presentation, gap identification, and process implementation to outcome achievement. However, it implicitly depends on a series of assumptions that link these steps together (Table 3). For instance, the assumption that presenting standards will allow gap identification presumes that standards will be thoroughly and uniformly understood, encompassing implicit as well as explicit meanings. In reality, standards are not always interpreted consistently (as shown in EP3), and institutions may focus on short term or superficial compliance (as seen in EO3). Furthermore, efforts to make standards less prescriptive often render them more abstract, further hindering a common understanding (Bórquez, 2023; Sjöström et al., 2019). The assumption that gap identification leads to process implementation presupposes favorable contextual conditions. Yet broader institutional and regulatory environments are not always conducive to such action (Taber et al., 2020). Moreover, if motivation to implement processes is low, schools may opt for selective compliance rather than full implementation. Finally, the expectation that executing a process yields the desired outcome overlooks unintended side effects. As suggested by EO1 and EO2, even when accreditation processes function as intended, both positive effects and adverse consequences can coexist.

**Table 3:** Core assumptions of the accreditation and associated emergent components

Stage	Primary Assumption	Secondary Assumption	Related Emergent Component(s)
a) Presentation of Standard			
b) Gap Identification	With the standard available, gaps can be detected	The standard will be clearly understood Institutions will actively seek out those gaps	EP3: Adapting to Evolving Accreditation Standards EO3: Strategic Short-Termism Under Pressure
c) Process Implementation	Detected gaps can trigger necessary processes	The conditions required to carry out the process are met There is sufficient motivation for process execution	EP1: Navigating Conflicting Accreditation Demands EP2: Strategic Control of Data Disclosure
d) Outcome Achievement	Implemented processes will yield the desired outcomes	The positive effects of the process will outweigh negatives.	EO1: Emotional Ambivalence Toward Accreditation EO2: Resource Intensification and Opportunity Costs
e) QA and QI Realization			

### Implication

This study suggests that both medical schools and accrediting organizations should take proactive steps to mitigate unintended consequences that could weaken accreditation’s positive functions. Recognizing institutions as CASs requires accepting that such emergent outcomes are inevitable within volatile, uncertain, and ambiguous environment (McKimm et al., 2023). Medical schools should therefore view accreditation as a continuous process of quality assurance and improvement rather than a single milestone. While quick, superficial fixes may help achieve accreditation in the short term, they leave institutions vulnerable to reverting to former practices (Cianciolo & Regehr, 2019). To ensure the sustainability of accreditation efforts, institutions require adaptive leadership (McKimm et al., 2023) capable of managing uncertainty and fostering organizational learning from emergent change (Van Aerde et al., 2023). They should also plan resource allocation to account for both one-time and recurring costs. Finally, institutions are encouraged to anticipate potential faculty burnout by treating it as critical feedback from the system, recognizing and rewarding participation, and providing resources to prevent and address burnout (Hoffmann, 2024).

Accreditation bodies are encouraged to cultivate trust with the schools they evaluate, recognizing them as CASs that thrive on self-organization, not rigid top-down control (Mennin, 2010). In doing so, they should allow institutions flexibility in interpreting standards, framing standards as shared “simple rules” (van Schaik et al., 2019) that enable local adaptation rather than prescriptive checklists. This flexibility must be balanced with effort to ensure that evaluators share a consistent understanding, thereby preventing biased assessments (Goldie, 2006; Greenfield et al., 2009). Although accreditation serves to validate program quality for external audiences, agencies should also foster a collaborative relationship with institutions (Crampton et al., 2019; Maccarrick et al., 2010). This involves providing constructive feedback and guidance, moving beyond a purely evaluative stance that can stifle the emergent innovations (Van Aerde et al., 2023). Such an approach also encourages transparency concerning areas requiring development (Taber et al., 2020).

Future research should continue to investigate the emergent components within medical schools. As the landscape of medical education evolves, accreditation standards and systems will likely adapt in response to these changes, shaping the nature of accreditation’s effects. This review also found that reported impacts tend to vary according to study context and design, indicating a need for more primary research that intentionally investigates emergent outcomes to advance understanding in this area. Importantly, although this study identified key planned and emergent components and proposed conceptual relationships among them, new primary research is essential to examine whether these relationships hold in practice, and if so, to what extent. Such investigations would provide stronger empirical foundations for the theoretical framework presented here. The planned and emergent theories developed in this study are dynamic constructs, open to refinement based on this future work. Lastly, while this review focused on institutional level dynamics, subsequent research could narrow its focus to students, faculty, or thematic areas such as social accountability and DEI (diversity, equity, and inclusion). Alternatively, research could broaden its scope to examine accreditation bodies and national education systems.

### Limitation

This study has several limitations. First, our analysis was limited to literature published in English. Given that accreditation is highly context-dependent, this may have excluded important perspectives from Low- and Middle-Income Countries (LMICs), where insightful reports might be published in local languages. However, a prior scoping review on UME accreditation, which did include non-English texts, found that 69% of publications were from high-income countries and none were from low-income countries (Tackett et al., 2019). This suggests that while our English-only focus is a constraint, our findings may not be excessively biased regarding income settings. Second, beyond geographic and linguistic variation, contextual variables such as the historical evolution of accreditation systems, the extent of brain drain, and whether a single or multiple accreditation agencies govern a jurisdiction could also significantly influence accreditation’s impact (Frenk et al., 2010; Voronovi et al., 2024). Third, our findings may be affected by a specific form of reporting bias. The studies included in this review likely did not have the explicit goal of investigating “emergent” outcomes. Consequently, it is probable that many emergent processes and outcomes were not reported at all, a bias that extends beyond the simple overrepresentation of positive findings. Fourth, the temporal evolution of accreditation’s impact was not accounted for in this review. Lastly, while we derived conceptual planned and emergent theories, our examination of process-outcome relationships is qualitative and inferential. Additional empirical work is required to confirm and quantify these theoretical linkages.

## Conclusion

This review highlights that accreditation should be conceptualized not as a fixed or solely procedural mechanism, but as a dynamic and evolving process, shaped by institutional agency and broader systemic conditions. While planned elements generally support quality enhancement, emergent phenomena may interfere with or limit these gains. Emotional fatigue, resource-related compromises, and contextually driven strategic behavior emerge as key unintended outcomes. Achieving accreditation’s dual mandate, quality assurance and improvement, will require medical schools and accrediting bodies to adopt a systems-based orientation that acknowledges complexity, promotes sustained participation, and builds reciprocal trust.

## Electronic supplementary material

Below is the link to the electronic supplementary material.

Supplementary Material 1

## Data Availability

No datasets were generated or analysed during the current study.
