# Supplementary material for: Beyond intentions: a critical narrative review of accreditation’s planned and emergent impact on medical schools
Source: Adv Health Sci Educ Theory Pract. 2026 Jan 24;31(4):1419–43. doi: 10.1007/s10459-026-10501-7 (PMC13553702; doi:10.1007/s10459-026-10501-7)
Supplement: Supplementary file 1 [file 10459_2026_10501_MOESM1_ESM.docx]

## Appendix 1: Detailed Search Strategies

## Ovid MEDLINE(R) ALL 1946 to November 20, 2024

Search Strategy:

| **#** | **Searches** | **Results** |
| --- | --- | --- |
| 1 | Schools, Medical/ | 28,621 |
| 2 | (medic* adj4 (school* or facult*)).mp. | 98,086 |
| 3 | (undergrad* adj4 med* ed*).mp. | 5,097 |
| 4 | ("med* ed*" adj4 ("year 1" or "year one" or "first year" or yr1 or "year 2" or "year two" or "second year" or yr2 or "year 3" or "year three" or "third year" or yr3 or "year 4" or "year four" or "fourth year" or yr4)).mp. | 109 |
| 5 | (student adj6 clerk*).mp. | 866 |
| 6 | or/1-5 | 101,301 |
| 7 | Accreditation/ | 15,452 |
| 8 | accredit*.mp. | 41,546 |
| 9 | regulat*.mp. | 2,970,628 |
| 10 | or/7-9 | 3,009,338 |
| 11 | 6 and 10 | 4,451 |

## Ovid Embase Classic+Embase 1947 to 2024 November 20

Search Strategy:

| **#** | **Searches** | **Results** |
| --- | --- | --- |
| 1 | medical school/ | 79,751 |
| 2 | (medic* adj4 (school* or facult*)).mp. | 150,896 |
| 3 | (undergrad* adj4 med* ed*).mp. | 5,699 |
| 4 | ("med* ed*" adj4 ("year 1" or "year one" or "first year" or yr1 or "year 2" or "year two" or "second year" or yr2 or "year 3" or "year three" or "third year" or yr3 or "year 4" or "year four" or "fourth year" or yr4)).mp. | 150 |
| 5 | (student adj6 clerk*).mp. | 1,127 |
| 6 | or/1-5 | 154,529 |
| 7 | accreditation/ | 42,267 |
| 8 | accredit*.mp. | 67,212 |
| 9 | regulat*.mp. | 3,618,555 |
| 10 | or/7-9 | 3,681,272 |
| 11 | 6 and 10 | 7,512 |

## EBSCOhost Research Databases – ERIC

Search Screen - Advanced Search

Expanders - Apply equivalent subjects

Search modes - Proximity

| **#** | **Query** | **Results** |
| --- | --- | --- |
| S10 | S6 AND S9 | 320 |
| S9 | S7 OR S8 | 52,142 |
| S8 | accredit* or regulat* | 52,142 |
| S7 | DE "Accreditation (Institutions)" | 6,914 |
| S6 | S1 OR S2 OR S3 OR S4 OR S5 | 8,074 |
| S5 | student N6 clerk* | 295 |
| S4 | med* ed* N4 ("year 1" or "year one" or "first year" or yr1 or "year 2" or "year two" or "second year" or yr2 or "year 3" or "year three" or "third year" or yr3 or "year 4" or "year four" or "fourth year" or yr4) | 25 |
| S3 | undergrad* N4 med* ed* | 227 |
| S2 | medic* N4 (school* or facult*) | 7,741 |
| S1 | DE "Medical Schools" OR DE "Medical School Faculty" | 3,854 |

## ProQuest Dissertations & Theses Global Closed Collection

noft((medic* NEAR/4 (school* OR facult*)) NEAR/8 (accredit* OR regulat*))

67 results

## Global Index Medicus (including all databases)

Title, abstract, subject:

(medical school* OR medical facult* OR undergrad* medical education) AND (accreditat* OR regulat*)

1726 results total

Database breakdown:

- LILACS (Americas) (993)
- WPRIM (Western Pacific) (476)
- IMSEAR (South-East Asia) (125)
- IMEMR (Eastern Mediterranean) (118)
- AIM (Africa) (14)

## Appendix 2: Characteristics of included studies

| Author | Year | Country / Region | Income Setting ^a^ | Study Design | Primary Data Source | Study Aim | Reported Impact of Accreditation ^b^ | | | | | |
| --- | --- | --- | --- | --- | --- | --- | --- | --- | --- | --- | --- | --- |
|  |  |  |  |  |  |  | PP | | PO | EP | | EO |
| Alrebish et al. | 2017 | Saudi Arabia | High | Qualitative | Interviews, Focus group, Documents | To examine the purposes, outcomes, and stakeholders' experiences of the accreditation of undergraduate medical schools in Saudi Arabia. |  |  | | ● |  | |
| Altıntaş et al. | 2024 | Turkey | Upper-Middle | Mixed-methods | Surveys, Documents | To present a self-evaluation of the accreditation activities of the Association for Evaluation and Accreditation of Medical Education Programs (TEPDAD), a medical education accreditation agency in Turkey. | ● | ● | |  |  | |
| Arja et al. | 2021 | Multi-country  ^c^ | Varied | Qualitative | Interviews | To investigate the perceptions of leaders and faculty at Caribbean medical schools regarding the impact of accreditation on their school's processes. | ● |  | |  | ● | |
| Arja, White, et al. | 2024 | Multi-country  ^c^ | Varied | Qualitative | Interviews | To investigate how accreditation influences CQI in Caribbean undergraduate medical education programs. | ● | ● | | ● |  | |
| Arja, Kumar, et al. | 2024 | Curaçao | High | Quantitative | Surveys | To explore the relationship between accreditation site visits and student satisfaction at Avalon University School of Medicine. |  | ● | |  |  | |
| Blouin et al. | 2018 | Canada | High | Qualitative | Interviews, Focus group discussions | To evaluate the impact of accreditation on the processes implemented at Canadian medical schools. | ● |  | |  | ● | |
| Chandran et al. | 2013 | United States | High | Descriptive case study | Documents | To describe how Stony Brook University School of Medicine used a LCME site visit to design a change management approach for institutional transformation and quality improvement | ● |  | |  |  | |
| Gaxiola-García et al. | 2020 | Mexico | Upper-Middle | Quantitative | Databases | To determine whether medical school accreditation status, institutional type (public vs. private), and geographic region are associated with performance in Mexico’s national medical residency admission exam | ● |  | |  |  | |
| Geffen et al. | 2014 | China | Upper-Middle | Descriptive case study | Documents | To describe the collaboration between China and Australia to establish a medical school accreditation process in China, based on international standards and guidelines | ● | ● | |  |  | |
| Gul et al. | 2022 | Pakistan | Lower-Middle | Qualitative | Focus group discussions | To explore the challenges faced by medical colleges in Pakistan and the coping strategies they used in implementing accreditation standards during the 2019 accreditation inspection by the Pakistan Medical Commission. |  |  | | ● |  | |
| Hedrick et al. | 2019 | United States | High | Mixed-methods | Surveys | To detail how U.S. medical schools are implementing CQI processes in response to new accreditation requirements from the LCME | ● |  | |  |  | |
| Hunt et al. | 2012 | United States | High | Quantitative | Documents | To determine the frequency of severe action decisions made by the LCME before and after the 2002 reformatting of accreditation standards |  |  | | ● |  | |
| Kassebaum, Cutler, et al. | 1997 | United States | High | Mixed-methods | Databases, Documents | To examine the influence of accreditation on educational change and reform in U.S. medical schools between July 1992 and June 1997 | ● | ● | |  |  | |
| Kassebaum, Eaglen, et al. | 1997 | United States | High | Mixed-methods | Databases, Documents | To examine the operational meaning of 48 accreditation standards for teaching, learning, and evaluation in U.S. medical schools and to determine the extent to which these standards were applied by both the schools and the on-site evaluators. |  |  | | ● |  | |
| Khan et al. | 2020 | Pakistan | Lower-Middle | Qualitative | Interviews, Focus group discussions | To identify the challenges facing the quality assurance and accreditation system for Basic Medical Education in Pakistan. |  |  | |  | ● | |
| Knollmann-Ritschel | 2017 | United States | High | Descriptive case study | Documents | To describe the perspectives and experiences of pathology course directors at the Uniformed Services University of the Health Sciences in preparing for an LCME accreditation visit under newly revised standards. |  |  | | ● |  | |
| Koepke et al. | 2020 | Canada | High | Qualitative | Focus group discussions | To explore the key drivers of social accountability in Canadian medical schools from the perspectives of medical school staff, students, and faculty. |  | ● | |  |  | |
| Li et al. | 2022 | China | Upper-Middle | Quantitative | Documents | To investigate the status of publicly available information from the 129 WCAME-accredited medical schools in China and to determine if there were significant differences among different types of universities. |  |  | | ● |  | |
| Muhtadi | 2013 | United States | High | Quantitative | Surveys | To assess the value of accreditation of undergraduate medical education programs in the U.S. by examining the perceived benefits and costs from the perspective of internal stakeholders |  | ● | |  | ● | |
| Onyura et al. | 2023 | Multi-country ^d^ | High | Qualitative | Interviews | To critically examine how paradoxical tensions shape evaluation practices in academic health science institutions pursuing accreditation. |  |  | | ● | ● | |
| Villamor & Reyes | 2016 | Philippines | Lower-Middle | Mixed-methods | Surveys, Documents | To describe the perspectives of stakeholders (faculty, students, graduates) on the impact of the accreditation process on the implementation of the medical curriculum. | ● |  | |  |  | |
| Roy et al. | 2020 | Canada | High | Quantitative | Databases | To explore the relationship between the accreditation cycle and student performance on the Medical Council of Canada Qualifying Examination Part I. |  |  | |  | ● | |
| Salcido | 2013 | United States | High | Qualitative | Interviews | To examine the early responses at three American medical schools regarding the implementation of new diversity accreditation standards introduced by the LCME in 2009. |  | ● | |  |  | |
| Schnabl | 2012 | United States | High | Mixed-methods | Interviews, Documents | To analyze how University of California (UC) medical schools addressed faculty diversity required by LCME accreditation standards while constrained by Proposition 209, which prohibits the use of race or gender in hiring. |  |  | | ● |  | |
| Scott et al. | 2020 | United States | High | Descriptive case study | Surveys, Documents | To describe the Independent Student Analysis Action (ISA²) process, designed to enhance student engagement and CQI throughout the LCME reaccreditation process. | ● |  | |  |  | |
| Tackett et al. | 2021 | Global | Varied | Quantitative | Databases | To examine the relationships between medical school accreditation variables and Educational Commission for Foreign Medical Graduates (ECFMG) certification for a global sample |  | ● | |  |  | |
| van Zanten et al. | 2012 | Multi-country  ^e^ | Varied | Quantitative | Databases | To investigate the relationship between medical school accreditation status and student performance on the USMLE for medical school graduates from Mexico and the Philippines. |  | ● | |  |  | |
| van Zanten et al. | 2015 | Global | Varied | Quantitative | Databases | To investigate the performance of international medical graduates on the USMLE based on the accreditation of their medical education programs. |  | ● | |  |  | |
| van Zanten et al. | 2022 | Global | Varied | Quantitative | Databases | To evaluate whether attendance at a medical school accredited by a WFME-recognized agency is associated with better USMLE first-attempt performance |  | ● | |  |  | |
| Ward et al. | 2018 | United States | High | Descriptive case study | Documents | To describe the implementation of a course review process for a CQI model at Meharry Medical College School of Medicine, to ensure compliance with LCME accreditation standards. | ● |  | |  |  | |
| White et al. | 2013 | Canada | High | Qualitative | Interviews | To explore the attitudes and experiences of leaders who were responsible for making rapid changes to a medical school curriculum in response to an adverse accreditation report. | ● | ● | |  | ● | |
| You et al. | 2024 | China | Upper-Middle | Quantitative | Databases | To examine the relationship between first-round accreditation and the performance of medical schools on medical licensing examinations in China, with a hypothesis that the impact of accreditation depends on existing performance gaps |  | ● | |  |  | |

^a^ Income setting classification follows the World Bank criteria relevant to the study period.

^b^ PP: Planned Processes, PO: Planned Outcomes, EP: Emergent Processes, EO: Emergent Outcomes

^c^ Caribbean region

^d^ Australia, Canada, Hong Kong, and the USA, all of which are High-Income Countries.

^e^ Mexico (Upper-Middle-Income Country) and the Philippines (Lower-Middle-Income Country)
